# Clinical and Molecular Insights in Erythropoiesis Regulation of Signal Transduction Pathways in Myelodysplastic Syndromes and β-Thalassemia

**DOI:** 10.3390/ijms22020827

**Published:** 2021-01-15

**Authors:** Sarah Parisi, Carlo Finelli, Antonietta Fazio, Alessia De Stefano, Sara Mongiorgi, Stefano Ratti, Alessandra Cappellini, Anna Maria Billi, Lucio Cocco, Matilde Y. Follo, Lucia Manzoli

**Affiliations:** 1 Department of Oncology and Hematology, IRCCS-Azienda Ospedaliero-Universitaria di Bologna, 40138 Bologna, Italy; sarah.parisi@alice.it (S.P.); carlo.finelli@unibo.it (C.F.); 2 Department of Experimental, Diagnostic and Specialty Medicine DIMES, Institute of Hematology “L. and A. Seràgnoli”, University of Bologna, 40138 Bologna, Italy; 3 Cellular Signalling Laboratory, Department of Biomedical and Neuromotor Sciences, University of Bologna, 40126 Bologna, Italy; antonietta.fazio2@unibo.it (A.F.); alessia.destefano3@unibo.it (A.D.S.); s.mongiorgi@unibo.it (S.M.); stefano.ratti@unibo.it (S.R.); alessandra.cappellini@unibo.it (A.C.); annamaria.billi@unibo.it (A.M.B.); lucio.cocco@unibo.it (L.C.); lucia.manzoli@unibo.it (L.M.)

**Keywords:** erythropoiesis, signal transduction, myelodysplastic syndromes, β-thalassemia, inositides

## Abstract

Erythropoiesis regulation is essential in normal physiology and pathology, particularly in myelodysplastic syndromes (MDS) and β-thalassemia. Several signaling transduction processes, including those regulated by inositides, are implicated in erythropoiesis, and the latest MDS or β-thalassemia preclinical and clinical studies are now based on their regulation. Among others, the main pathways involved are those regulated by transforming growth factor (TGF)-β, which negatively regulates erythrocyte differentiation and maturation, and erythropoietin (EPO), which acts on the early-stage erythropoiesis. Also small mother against decapentaplegic (SMAD) signaling molecules play a role in pathology, and activin receptor ligand traps are being investigated for future clinical applications. Even inositide-dependent signaling, which is important in the regulation of cell proliferation and differentiation, is specifically associated with erythropoiesis, with phospholipase C (PLC) and phosphatidylinositol 3-kinase (PI3K) as key players that are becoming increasingly important as new promising therapeutic targets. Additionally, Roxadustat, a new erythropoiesis stimulating agent targeting hypoxia inducible factor (HIF), is under clinical development. Here, we review the role and function of the above-mentioned signaling pathways, and we describe the state of the art and new perspectives of erythropoiesis regulation in MDS and β-thalassemia.

## 1. Introduction

Erythropoiesis is an essential process that is finely regulated. All stages are controlled by several molecules and several signal transduction pathways are implicated. For instance, the transforming growth factor (TGF)-β family negatively regulates the erythrocyte differentiation and maturation, whilst erythropoietin (EPO) is the main regulator of early-stage erythropoiesis. Ineffective erythropoiesis may cause anemia and is particularly relevant in myelodysplastic syndromes (MDS) and in homozygous β-thalassemia, where small mother against decapentaplegic (SMAD) signaling is altered and activin receptor ligand traps have been shown to correct the hyperactivation of SMAD2/3. New clinical approaches to counteract anemia and support erythropoiesis are based on Roxadustat, a new erythropoiesis stimulating agent targeting hypoxia inducible factor (HIF). Finally, some data also hint at a relevant role for phospholipase C and PI3K in erythropoiesis regulation. This review will focus on these pathways and describe their importance in preclinical studies and clinical applications, because they are the target of new therapeutic agents which have proved to be highly effective in improving anemia associated with MDS, β-thalassemia and chronic kidney disease.

## 2. Targeting Transforming Growth Factor (TGF)-β Signaling to Improve Erythropoiesis

### 2.1. Effective and Ineffective Erythropoiesis

**Figure 1:**
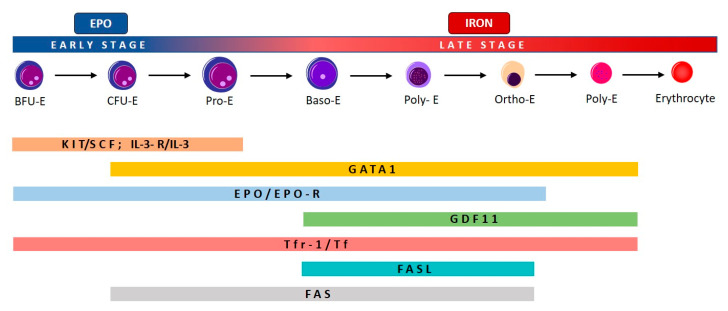
Schematic representation of erythroid development and time-dependent expression of a complex network of transcription factors involved in the process. EPO-dependent and iron-dependent stages of erythropoiesis are indicated. Differentiation, maturation, and proliferation of erythroid progenitors are regulated by several molecules according to different stages. Stem cell factor (SCF), interleukin-3 (IL-3) and their receptors progressively disappear during erythroid maturation, after the Pro-E stage. GATA1 promotes erythropoiesis and increases the EPO receptor (EPO-R) expression, which is maintained until the Ortho-E stage. In turn, EPO is the main regulator of early-stage erythropoiesis, after the BFU-E stage. Other molecules are regulators of iron metabolism, such as transferrin (Tf) and its cell receptor (Tfr), growth/differentiating factor 11 (GDF11) and members of the transforming growth factor (TGF)-β family, which negatively regulate erythrocyte differentiation and maturation from the early stages (Tf/Tfr-1) to the late stages (Poly-E). Finally, the binding between FAS and FAS ligand (FASL) triggers the apoptosis of immature erythroid cells, acting from the CFU-E to the Ortho-E stages.

The process of erythropoiesis in humans is divided in two parts: the first is EPO-dependent and the second is iron-dependent (Figure 1). Erythropoiesis occurs in erythroblastic islands, anatomic niches composed of a central macrophage surrounded by erythroid cells at various stages of maturation: erythroid burst-forming units (BFU-E), erythroid colony-forming units (CFU-E), erythroid progenitors (proerythroblasts and erythroblasts) [1].

As depicted in Figure 1, proliferation, differentiation, and maturation of erythroid progenitors is controlled by several molecules which act at different stages of erythropoiesis. Stem cell factor (SCF) and interleukin 3 (IL-3) target hematopoietic stem cells (HSC), and their receptors gradually disappear during erythroid maturation. GATA1 promotes erythropoiesis during several stages, because it enhances the expression of the anti-apoptotic gene of the BCL family, *Bcl-x*, and of *EPO-R*, and stimulates the synthesis of hemoglobin [2].

The later iron-dependent phase of erythropoiesis is regulated both by transferrin and its cell receptor, and by growth/differentiating factor 11 (GDF11) and by other members of the TGF-β family, which negatively regulate erythrocyte differentiation and maturation [3].

EPO is the main regulator of early-stage erythropoiesis: the binding of EPO to EPO-R, located on the surface of the erythroid precursors, causes the activation of JAK2 which, in turn, induces the phosphorylation and activation of STAT5, with a subsequent activation of erythroid antiapoptotic genes and enhancement of survival and proliferation of erythroid progenitors [4]. The mechanism that regulates the expansion of erythropoiesis in response to a possible increase in demand (e.g., hypoxia, hemorrhage, or hemolysis) is EPO-dependent and is based on FAS and the FAS ligand (FASL), which belong to the tumor necrosis factor (TNF) family, whose binding activates the caspase cascade and, consequently, cell apoptosis. If the production of erythrocytes is adequate, this mechanism enhances the apoptosis of immature cells, thus limiting the expansion of erythropoiesis. On the other hand, if the need for erythrocytes, and consequently the production of EPO, increase, then apoptosis is reduced, because EPO stimulates the production of heat shock protein 70 (HSP70) which protects GATA1 from cleavage by FAS/FASL and caspase cascade [2].

**Figure 2:**
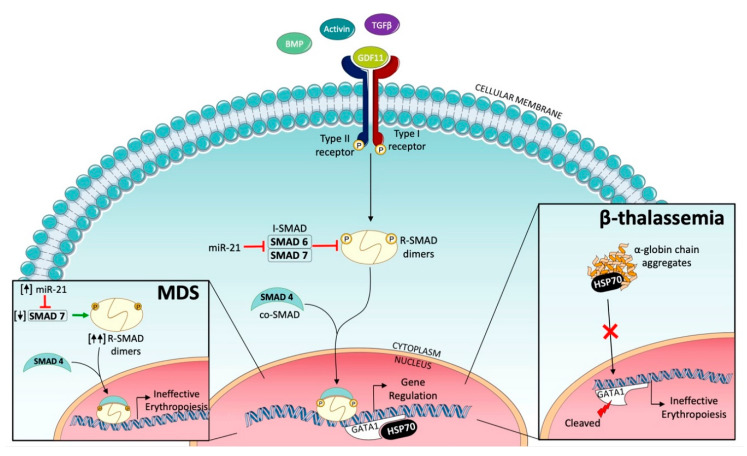
Erythropoiesis regulation by TGF-β superfamily signaling in physiologic and pathologic conditions, particularly in myelodysplastic syndromes (MDS) and β-thalassemia. Following ligand binding to the type II receptor, the type I receptor is recruited and phosphorylated. TGF-β induces the phosphorylation of regulatory SMADs (R-SMADs), SMAD2 and 3, to form the complex R-SMAD/SMAD4, which modulates the expression of target genes. MDS patients display an overactivation of SMAD2/3 signaling due to the altered expression of mir-21 and SMAD7, which in turn induce ineffective erythropoiesis. In β-thalassemia, aggregates of α-globin sequester heat shock protein 70 (HSP70), inhibiting its translocation to the nucleus and protection of GATA1 from cleavage, leading to ineffective erythropoiesis.

Late-stage erythropoiesis is mainly regulated by TGF-β superfamily signaling. TGF-β receptors are a superfamily of serine/threonine kinase receptors classified into three types: type I, type II, and type III. There are seven type I receptors, termed activin-like receptors (ALK1-7), five type II receptors, and one type III receptor. The TGF-β signaling pathway involves ligand binding to the type II receptor, with consequent recruitment and phosphorylation of the type I receptor. Activin type IIA and B receptors belong to the type II family receptors [3]. TGF-β receptor ligands are a group of polypeptide growth factors which include TGF-β, activins, bone morphogenetic proteins (BMPs), and growth differentiation factor (GDF-11). These cytokines play an important role in the regulation of hematopoiesis within the hematopoietic stem cell niche, and in particular activins and GDF-11 exert an inhibitory activity on late-stage erythropoiesis, distinct from EPO activity on early-stage erythropoiesis. The subsequent stages of ligand binding and phosphorylation of type I and II receptors are: phosphorylation of regulatory SMADs (R-SMADs) SMAD2 and SMAD3; binding of an R-SMAD pair to the co-SMAD protein SMAD-4; translocation of this trimeric SMAD complex to the nucleus; binding to chromatin; and modification of the expression of target genes (Figure 2) [5]. SMAD family proteins, named for their similarity to the Drosophila gene mothers against decapentaplegic (MAD), are encoded by a gene located on chromosome 15q22.33, and are signal transducers and transcription modulators of multiple signaling pathways. The activity of this pathway is regulated by the feedback of inhibitory SMADs (I-SMADs) such as SMAD6 and SMAD7 [6]. Furthermore, TGF-β superfamily ligands can activate other pathways, such as MAP kinases, as well as PI3K/AKT. In healthy organisms, TGF-β signaling has a myelosuppressive activity and inhibits erythroid differentiation by inducing apoptosis and cell cycle arrest in erythroblasts [7]. During normal erythroid maturation, both the suppression of TGF-β signaling, such as reduced GDF expression and the stimulation by erythropoietin, occur in parallel [8].

### 2.2. Ineffective Erythropoiesis in Myelodysplastic Syndromes (MDS)

MDS are a heterogeneous group of clonal hematopoietic stem cell disorders characterized by dysplastic bone marrow morphology, impaired hematopoiesis resulting in peripheral cytopenia, and a variable risk of leukemic evolution [9]. The majority of MDS patients present anemia, which is often macrocytic and is usually caused by ineffective erythropoiesis, which is particularly accentuated in a subgroup of MDS, called MDS with ring sideroblasts (MDS-RS) according to the 2016 World Health Organization (WHO) classification [10], which are characterized by the presence of iron deposition in the mitochondria in the form of mitochondrial ferritin and by the somatic mutation of SF3B1, a gene encoding splicing factor [11,12].

Several studies showed that the TGF-β superfamily signaling is altered in MDS [13]. First of all, it was shown that MDS patients showed a higher number of bone marrow cells with activated phosphorylated SMAD2 (p-SMAD2) and a greater intensity of p-SMAD staining, compared to normal controls, and that therefore SMAD2 is constitutively activated and overexpressed in the hematopoietic cells of MDS patients. Furthermore, erythropoiesis was enhanced in vitro in MDS bone marrow cells if the activation of SMAD2 was counteracted by inhibition of the type I receptor ALK5 [14]. Subsequently, the cause of the overactivation of SMAD2/3 in MDS hematopoietic cells was investigated, and it was shown that the levels of SMAD7 were markedly reduced in MDS marrow cluster of differentiation 34+ (CD34+) cells compared to normal healthy controls [15]. SMAD7 is an important negative-feedback regulator of the TGF-β superfamily signaling, because it can associate with type I receptor ALK5 and interfere with interactions between ALK5 and SMAD2/3, thereby negatively affecting the activity of the SMAD2/3 pathway. Furthermore, SMAD7 has been shown to promote hematopoietic stem cell self-renewal in vivo [16]. In fact, in the hematopoietic cells of MDS patients, the reduced expression of SMAD7 was associated with an overactivation of SMAD2/3 signaling, even in the presence of low TGF-β concentrations. In addition, Galunisertib, an ALK5 inhibitor, was found to inhibit TGF-β-mediated SMAD2/3 hyperactivation and to stimulate hematopoiesis in bone marrow cells from MDS patients [15]. Moreover, in a phase II study, treatment with the ALK5 inhibitor Galunisertib was associated with erythroid response, according to the IWG 2006 response criteria, in 24.4% of patients with lower- and intermediate-risk MDS [17].

A subsequent study shed light on the cause of reduced SMAD7 activity in MDS patients. In fact, SMAD7 inhibition is caused by the increased levels of microRNA-21 (miR-21) found in the bone marrow cells of MDS patients, compared to a group of age-matched healthy controls [18]. miR-21 is upregulated in many diseases and is considered a key switch in the inflammatory response [19]. Furthermore, it has been shown that the 3′-UTR of the *SMAD7* gene contains a highly conserved, putative binding site for miR-21, and that miR-21 directly binds to the 3’-UTR of the *SMAD7* gene and reduces its expression in hematopoietic cells. Finally, it was observed that the in vitro treatment with a miR-21 inhibitor increased the expression of SMAD7 and also erythroid colony formation in samples obtained from MDS patients [18].

These results indicated that the reduced activity of SMAD7, caused by the increased levels of miR-21, was the cause of the overactivation of SMAD2/3 and of the consequent ineffective erythropoiesis in MDS, and that this pathway could be a potential therapeutic target to correct anemia in MDS patients (Figure 2).

### 2.3. Ineffective Erythropoiesis in β-Thalassemia

In β-thalassemia, a mutation in the *β-globin* gene leads to an imbalance between α- and β-globin chains, and consequently to ineffective erythropoiesis that results in impaired differentiation of maturing erythroblasts at the polychromatic and orthochromatic phase [20]. Anemia is caused by several pathogenetic mechanisms [21]. On the one hand, α-globin aggregates sequester cytosolic heat shock protein 70 (HSP70) and prevent its nuclear translocation and the protection of erythroid transcription factor GATA-binding factor 1 (GATA1) from cleavage (Figure 2). On the other hand, the excess of α-globin stimulates the formation of radical oxygen species (ROS) (whose formation is also enhanced by the iron overload) which activate GDF11, that in turn activates the SMAD2/3 inhibitory pathway, resulting in inhibition of erythroid differentiation [22].

## 3. Targeting SMAD2/3 Signaling to Correct Ineffective Erythropoiesis

### 3.1. Activin Receptor Ligand Traps

A class of drugs, defined as activin receptor ligand traps, is capable of correcting the hyperactivation of SMAD2/3, by trapping SMAD2/3 pathway ligands other than TGF-β. These drugs are effective in the treatment of anemia associated with MDS and other hematological diseases [23,24]. Sotatercept (ACE-011) is a ligand-trapping fusion protein containing the extracellular domain of activin receptor type IIA (ActRIIA), attached to the Fc domain of human IgG1 (ActRIIA-Fc), which binds activins A and B, GDF8 and GDF11, and some BMPs (BMP6, BMP7, and BMP10) with a range of affinities, reflecting the binding profile of native ActRIIA [25]. Luspatercept (ACE-536), structurally similar to Sotatercept, is a ligand-trapping fusion protein containing a modified extracellular domain of activin receptor type IIB (ActRIIB) attached to the Fc domain of human IgG1 (modified ActRIIB-Fc), which, similarly to Sotatercept, binds GDF8, GDF11, and activin B, but differs from Sotatercept and from native ActRIIA due to a reduced affinity for activin A [26,27]. Neither Luspatercept nor Sotatercept bind TGF-β1, TGF-β2, or TGF-β3.

### 3.2. Activin Receptor Ligand Traps: Preclinical Studies

Both Luspatercept and Sotatercept demonstrated their efficacy in preclinical models using murine analogues—RAP-536 for Luspatercept and RAP-011 for Sotatercept—in which the human IgG1 domain was replaced by its mouse IgG2 counterpart. RAP-536 proved to be effective in increasing erythropoiesis in normal mice, rats, and monkeys by enhancing maturation of late-stage erythroblasts, and was also able to reduce ineffective erythropoiesis and anemia in a mouse model of MDS, characterized by ineffective erythropoiesis, by inhibiting the overactivation of SMAD2/3 [26]. RAP-536 was also shown to inhibit the overactivation of SMAD2/3 signaling, to improve anemia, and to decrease extramedullary erythropoiesis in a mouse model of β-thalassemia, also characterized by impaired erythroid maturation and ineffective erythropoiesis [28]. RAP-011 also proved to be effective in improving erythroid maturation and anemia and in reducing the spleen size and iron overload in a mouse model of β-thalassemia [29].

### 3.3. Activin Receptor Ligand Traps: Clinical Evaluation

Sotatercept was initially used in healthy post-menopausal women for the prevention of osteoporosis, due to its ability to increase bone mineral density, and in treated women it was observed, in addition to an increase in bone formation and a decrease in bone resorption, a sustained increase in hemoglobin level [30,31]. Luspatercept, also used in healthy post-menopausal women, showed similar results [32]. Sotatercept was also used in a phase IIA trial in multiple myeloma, showing not only anabolic improvements in bone mineral density and bone formation, but also a durable increase in hemoglobin levels [33]. Even in patients with chemotherapy-induced anemia, Luspatercept has been shown to be able to determine a significant increase in the hemoglobin level [34]. Subsequently, on the basis of encouraging preclinical results, both Sotatercept and Luspatercept have been assessed in patients with MDS and β-thalassemia [35].

### 3.4. Clinical Studies in MDS

Agents that interfere with TGF-β signaling were used in several clinical studies in MDS (Table 1).

In an open-label, multicenter, dose-ranging, phase II study, 74 patients with low- or intermediate-1-risk MDS and transfusion-dependent anemia were treated with Sotatercept, administered once every three weeks, subcutaneously, at 0.1, 0.3, 0.5, 1.0 or 2.0 mg/kg. The primary efficacy endpoint was the rate of hematological erythroid improvement (HI-E), according to the IWG 2006 criteria. A total of 36 of 74 patients (49%) achieved HI-E; 30 of 51 (59%) patients with ≥5% ring sideroblasts achieved HI-E, compared to 4/18 responders (22%) among patients with <15% ring sideroblasts (*p* = 0.0080). As for treatment-emergent adverse events (TEAs), grade 3–4 TEAs were observed in 25 cases, i.e., 34% [36].

Luspatercept was firstly tested in MDS patients participating in a phase II, multicenter, open-label, dose-finding study (PACE-MDS). Eligible patients had low- or intermediate-1-risk of non-proliferative chronic myelomonocytic leukemia and showed a transfusion-dependent anemia. Patients were treated with Luspatercept subcutaneously, once every 21 days, at dose concentrations ranging from 0.125 mg/kg to 1.75 mg/kg bodyweight, for five doses (over a maximum of 12 weeks). Patients in the expansion cohort were treated with 1.0 mg/kg Luspatercept; dose titration up to 1.75 mg/kg was allowed, and patients could be treated with Luspatercept for a maximum of five years. The primary endpoint was the proportion of patients achieving modified IWG-defined HI-E. A total of 58 patients were enrolled, with 27 patients enrolled in the dose-escalation cohorts (0.125–1.75 mg/kg) and 31 patients in the expansion cohort (1.0–1.75 mg/kg). Thirty-two (63%) out of 51 patients receiving higher dose Luspatercept concentrations (0.75–1.75 mg/kg) achieved HI-E, versus two (22%) out of nine receiving lower dose concentrations (0.125–0.5 mg/kg). Interestingly, response to Luspatercept was more frequent and more robust in patients with ring sideroblasts ≥15% (69% achieved IWG HI-E) or SF3B1 mutations (HI-E: 77%). The safety profile was favorable: only three treatment-related grade 3 adverse events were observed in one patient each [37].

Given that Luspatercept, as compared with Sotacercept, showed positive clinical results, a lower incidence of side effects and a narrower in vitro activity profile, it was selected and tested in MDS with ringed sideroblasts (in which Luspatercept had shown a greater efficacy) in a phase III, randomized, double-blind, placebo-controlled study (the MEDALIST trial) [38]. The main inclusion criteria were: MDS with ring sideroblasts (according to the 2016 WHO classification, i.e., either ≥15% ring sideroblasts or ≥5% ring sideroblasts with Splicing factor 3B subunit 1 (SF3B1) mutation); IPSS-R risk very low; low, or intermediate, transfusion-dependent anemia; and a disease refractory or unlikely to respond to ESAs (i.e., endogenous EPO levels >200 U/l or intolerance to ESAs). Of the 229 patients enrolled, 153 were randomly assigned to receive Luspatercept (at a dose of 1.0 up to 1.75 mg per kg of body weight, administered subcutaneously every three weeks) and 76 received a placebo; the baseline characteristics of the patients were balanced. The primary end point was transfusion independence for ≥8 weeks during weeks 1–24, and the key secondary end point was transfusion independence for ≥12 weeks, assessed during weeks 1–24 and weeks 1–48. As to primary end point, transfusion independence for ≥8 weeks was achieved in 38% of the patients in the Luspatercept group, as compared with 13% of those in the placebo group (*p* < 0.001). Furthermore, a higher percentage of patients in the Luspatercept group than in the placebo group met the key secondary end point (28% vs. 8% for weeks 1–24; *p* < 0.001; and 33% vs. 12% for weeks 1–48; *p* < 0.001). The median duration of the longest single period of transfusion independence was 30.6 weeks in the Luspatercept group versus 13.6 weeks in the placebo group. Overall, 65 patients (42%) of the Luspatercept arm and 34 (45%) of the placebo arm showed grade 3–4 adverse events. The most common Luspatercept-associated adverse events were fatigue, diarrhea, asthenia, nausea, and dizziness, but the incidence of adverse events decreased over time. In summary, Luspatercept significantly reduced the transfusion burden in a large percentage of MDS patients with ring sideroblasts who were refractory or unlikely to respond to ESAs and was mainly associated with low-grade adverse events.

### 3.5. Clinical Studies in β-Thalassemia

Activin receptor ligand traps are also effective in treating anemia associated with β-thalassemia. In fact, ineffective erythropoiesis, with hyperplasia of medullary immature erythroid cells and peripheral anemia, is also present in β-thalassemia and is caused by genetically derived hemoglobin defects. Following the results obtained in preclinical studies [28,29], activin receptor ligand traps have been assessed in anemic patients with β-thalassemia (Table 1).

Firstly, 64 patients were enrolled in an open-label, non-randomized, uncontrolled study that consisted of a 24-week dose-finding followed by an expansion stage and a subsequent five-year extension stage. Of the 64 patients, 33 had non-transfusion-dependent thalassemia (NTDT), while the other 31 had transfusion-dependent thalassemia (TDT) [39]. Patients were administered 0.2 to 1.25 mg/kg Luspatercept subcutaneously every 21 days for ≥5 cycles (dose-finding stage) and 0.8 to 1.25 mg/kg (expansion cohort and five-year extension). The primary end point was erythroid response, defined as a hemoglobin increase of ≥1.5 g/dL for ≥14 consecutive days for NTDT patients or ≥20% decrease in transfusion need over a 12-week period for TDT patients. Eighteen patients with NTDT (58%) receiving higher dose levels of Luspatercept (0.6–1.25 mg/kg) obtained a ≥1.5 g/dL increase in hemoglobin level over ≥14 days, and 26 (81%) TDT patients achieved a ≥20% reduction in transfusion requirement [39]. These findings led to a subsequent randomized, double-blind, phase III trial (BELIEVE study), in which 336 adult patients with TDT were enrolled [40]. The patients were assigned, in a 2:1 ratio, to receive the best supportive care plus Luspatercept (at a dose of 1.00 to 1.25 mg per kg of body weight) or a placebo, for at least 48 weeks. The primary end point was the percentage of patients who had ≥33% reduction in the transfusion burden during weeks 13–24, plus a ≥2 red cell unit reduction over this 12-week interval. Other efficacy end points included: reduction in the transfusion burden during any 12-week interval and results from iron studies. A total of 224 patients received Luspatercept, and 112 received the placebo. The primary end point was achieved in 21.4% versus 4.5% of patients in the Luspatercept and placebo arms, respectively (*p* < 0.001). The difference between the two groups was also significant for the other efficacy end points: a ≥33% reduction in transfusion need during any 12-week interval (70.5% vs. 29.5%); a ≥50% reduction in transfusion burden during any 12-week interval (40.2% vs. 6.3%), and a significant reduction in serum ferritin levels at week 48. The median longest duration of response was 104 days or 98 days among patients who achieved a ≥33% or ≥50% transfusion reduction, respectively. Most patients (80.4%) in the Luspatercept group who achieved the primary end point showed more than one distinct episode of response. The safety of Luspatercept was consistent with previous experience in this and other patient populations.

## 4. Targeting HIF: Clinical Development of Roxadustat, a New Erythropoiesis Stimulating Agent

Anemia in MDS is the major symptom, and is primarily due to ineffective erythropoiesis. Erythropoiesis-stimulating agents, such as recombinant EPO, are currently the first line of therapy in low-risk MDS patients who develop transfusion-dependent or symptomatic anemia. However, response to erythropoiesis-stimulating agents (ESAs) is not always satisfactory in terms of transfusion independence, and most patients are expected to lose response during their medical history. Moreover, ESA resistance mechanisms in MDS are only partially understood, but in vitro studies have indicated that there could be a defective transduction pathway mediated by ESAs [41,42,43].

HIF (hypoxia inducible factor) is a heterodimeric transcription factor involved in the production of EPO in response to hypoxia, and its activity is regulated by hypoxia inducible factor prolyl hydrolase domain (HIF-PHD) enzymes. These enzymes regulate the stability of the alfa-subunit of HIF and maintain a balance between the degree of hypoxia and HIF activity, thus influencing EPO production [44].

HIF transcription factors consist of two subunits, an oxygen-sensitive α-subunit and a constitutively expressed β-subunit. The α subunit is degraded in the presence of molecular oxygen; whereas in hypoxic conditions, it translocates to the nucleus and forms a heterodimer with HIF-β, thus activating HIF-1 and -2 gene transcription.

HIF-2 is responsible for the regulation of EPO production and iron uptake and utilization, and it enhances erythroid maturation and proliferation in the bone marrow [45]. Moreover, HIF-2 is able to increase EPO receptor expression on bone marrow.

Renal EPO-producing cells (EPCs) are oxygen sensor cells able to enhance EPO production through HIF-2 signaling pathways, in response to changes in tissue pO_2_. EPO synthesis then increases erythropoiesis and iron demand in the bone marrow.

Regarding iron metabolism, HIF-2 is able to increase the transcription of Divalent Metal Transporter 1 (DMT1) and Duodenal Cytochrome B (DCYTB), thus promoting iron uptake. Moreover, HIF-2 is able to suppress hepcidin transcription and to increase iron availability [45].

Roxadustat is an oral bioavailable inhibitor of the hypoxia inducible factor prolyl hydrolase (HIF-PHD) currently under investigation to treat anemia in chronic kidney disease and in MDS. Roxadustat inhibits the degradation of HIF-alfa, thus enhancing erythropoiesis in response to hypoxia [46]. Among patients with advanced chronic kidney disease (CKD), anemia is due to multiple mechanisms: decreased EPO production, iron deficiency, blood loss, inflammation, and decreased survival of erythrocytes.

Roxadustat was evaluated in a phase II, randomized, placebo-controlled study in 117 patients with CKD stage 3–4, nondialysis-dependent and anemia. Patients received four different doses of the drug (0.7, 1.0, 1.5 or 2 mg/kg) using different schedules (twice weekly—BIW, or three times weekly—TIW) or placebo (randomization 3:1).

A total of 293 subjects were screened and 117 were finally randomized to receive Roxadustat (*n* = 88) or the placebo (*n* = 29). Of the patients treated with Roxadustat, 68 (88.6%) were evaluable for efficacy, as were 26 (92.2%) who received placebo. Hemoglobin response rates (response was defined as a minimum increase of 1 g/dL of hemoglobin levels at any time of treatment) were 13% in the placebo group, 30 and 58% in the Roxadustat 0.7 mg/kg BIW and TIW groups, 60 and 40% in the 1.0 mg/kg BIW and TIW groups, 80 and 91% in the 1.5 mg/kg BIW and TIW groups, and 100% in both 2.0 mg/kg groups, respectively. The study concluded that patients who received Roxadustat had a significantly higher response rate at any dosage or schedule, compared to patients who received the placebo, and the rates of response were dependent on the Roxadustat dosage. The drug proved to be safe, and no difference in serious adverse events rate in the Roxadustat group versus the placebo group was evidenced [47]. Even correlative biological studies were conducted, in order to evaluate the effect of Roxadustat on EPO levels and iron metabolism.

In placebo subjects, EPO levels remained unchanged post-dose from baseline; for patients who received Roxadustat 1.0 mg/kg BIW, EPO levels reached the maximum increase on day 1 after 10 h post-dose (median 113 IU/L) and returned to baseline levels within 24−48 h. Less data were available with the other dose regimens. Authors concluded that EPO levels increased with dose but not frequency. As for iron metabolism, serum hepcidin levels decreased significantly during treatment with Roxadustat 1.5 and 2.0 mg/kg: mean hepcidin levels were both significantly decreased from baseline (−150 ± 89.5, *p* = 0.048, and −225 ± 192 ng/mL, *p* = 0.0013, respectively) compared with the placebo group (−17.8 ± 114 ng/mL) [47].

A subsequent phase II, randomized, dose-ranging study was conducted by Provenzano R et al. [48] on dialysis-dependent CKD patients treated with EPO. The aim of that study was to evaluate the efficacy of Roxadustat in terms of hemoglobin level increases of at least 0.5 g/dL from baseline by the end of week six of treatment, and to establish the optimal dosage of the investigational drug. A total of 144 patients were randomized (randomization 3:1) to receive Roxadustat or epoetin alfa; 125 patients resulted to be evaluable for efficacy. In the first part of the study, 54 patients were treated for six weeks (41 Roxadustat 1.0, 1.5, 1.8, 2.0 mg/kg thrice weekly, and 13 epoetin alfa). Patients treated with Roxadustat 1.0 mg/kg had a hemoglobin response comparable to that of epoetin alfa-treated patients (44% versus 33%). Intention-to-treat analysis showed that 63% of all Roxadustat participants had a response, versus 31% of epoetin alfa participants (*p* = 0.06).

In part 1 of the study, patients receiving Roxadustat had an overall mean increase in hemoglobin of 0.3 g/dL from baseline, compared to a decrease of 1 g/dL in patients who continued EPO treatment (*p* = 0.04). In part 2 of the study, 90 patients were treated for 19 weeks (67 Roxadustat 1.8 mg/kg, 6 patients Roxadustat at the other schedules, and 23 epoetin alfa). For Roxadustat overall, 31 out of 61 (51%) patients who were evaluable for efficacy achieved hemoglobin levels ≥11.0 g/dL over the last four weeks of the 19-week treatment period, versus 8 out of 22 (36%) epoetin alfa-treated patients. The safety profiles of Roxadustat and EPO analogs were comparable. In conclusion, phase II studies in CKD patients demonstrated that Roxadustat is effective in increasing and maintaining hemoglobin levels. Moreover, these studies also proved its efficacy in increasing endogenous EPO levels and in suppressing hepcidin, thus affecting iron utilization [47,49].

In 2019, Chen N et al. [50] published the results of their phase III, double-blind, trial on anemic, non-dialysis CKD patients. A total of 154 patients were randomly assigned to receive Roxadustat (*n* = 102) or a placebo (*n* = 52) for eight weeks; 131 patients were evaluable for efficacy (87 in the Roxadustat group and 44 in the placebo group). Patients treated with Roxadustat had an increase in hemoglobin levels of 1.9 ± 1.2 g/dL versus a decrease of 0.4 ± 0.8 g/dL in the placebo group. After nine weeks of treatment, 85 out of 101 patients (84%) in the Roxadustat group and none of the 50 patients in the placebo group had a hemoglobin response (increase of ≥1.0 g/dL from baseline). In the subsequent 18-week-open-label phase, 87 patients from the previous Roxadustat group and 44 patients from the previous placebo group (*n* = 131) received Roxadustat, and 98 completed the open-label phase. Among patients who had previously received Roxadustat in the randomized phase, hemoglobin levels remained stable (overall increase from baseline of 1.9 ± 1.3 g/dL). Moreover, 84% of patients achieved a hemoglobin level ≥11.0 g/dL, and 62 patients (73%) achieved a hemoglobin level ≥ of 10.0 g/dL. Among patients who had previously received the placebo, the mean hemoglobin increase from baseline was 2.0 ± 1.5 g/dL after crossover. Most (72%) of the patients achieved a hemoglobin level ≥11.0 g/dL, and 37 patients (86%) achieved a hemoglobin level ≥10.0 g/dL. The study proved that Roxadustat was more effective than the placebo in correcting anemia (increase in hemoglobin levels of 1.9 ± 1.2 g/dL vs. decrease of 0.4 ± 0.8 g/dL in the placebo group) and that this response was maintained during the 18 month-open-label phase. The previously described effect in reducing hepcidin levels, associated with an increase in iron availability, was confirmed.

## 5. Targeting Inositide-Dependent Signal Transduction Pathways

Phosphoinositides (PIs) form just a minor portion of the total phospholipid content in cells, but they are significantly involved in cancer development and progression [51]. PI metabolism includes phospholipases, a family of phospholipid metabolizing enzymes that catalyze the production of inositol-1,4,5-trisphosphate (IP3) and diacylglycerol (DAG) as secondary messengers implicated in several downstream pathways [52]. Four major families of phospholipases have been identified so far: A, B, C and D. Among all the phospholipases C (PLCs), nuclear PLCβ1 is an essential enzyme that regulates cell proliferation and differentiation in several systems [53].

In hematopoiesis, and particularly in MDS, PLCβ1 is associated with both myelopoiesis and erythropoiesis, although with opposite effects [54,55,56].

When induced by demethylating agents, such as Azacitidine, nuclear PLCβ1 expression is induced, and this can promote MDS myeloid differentiation. On the contrary, PLCβ1 is a negative regulator of erythropoiesis; MDS cells obtained from lower-risk MDS during EPO therapy specifically downregulate nuclear PLCβ1 expression and activate the PI3K/Akt/PLCγ1 pathway, thus resulting in apoptosis and a low proliferation rate of MDS cells [57,58].

Other studies have also demonstrated the importance of nuclear inositides in MDS patients treated with Lenalidomide, an immunomodulatory drug that is particularly effective in MDS patients showing a specific deletion of chromosome 5q [del(5q)]. These patients are usually at lower risk of AML evolution, but are characterized by ineffective erythropoiesis, which can be restored by Lenalidomide. Nuclear PLCβ1, along with its downstream target PKCα, is implicated in the erythroid differentiation induced by Lenalidomide, and its localization seems to be essential. In fact, in del(5q) MDS patients responding to Lenalidomide, PLCβ1 expression is specifically increased only in the cytoplasm of the del(5q) cells, whereas, in the same subpopulation, PKCα translocates to the nucleus [59].

Ineffective eythropoiesis is associated with the decreased functionality of erythroid progenitors and can also be linked to a defective platelet production, which is also linked to inositide signaling. In fact, EPO exhibits in vitro megakaryocytic potential [60], which can be driven by PI metabolism. Indeed, PIs are strongly involved in different aspects of platelet production and activation. The well-known signaling pathway involving PLC and its substrate phosphatidylinositol (4,5) bisphosphate (PIP2) to generate the second messengers IP3 and DAG is critical in platelet activation. Even PI3K is implicated: class I PI3K (particularly PI3Kβ isoform) and its product PtdIns(3,4,5) trisphosphate (PIP3) are indeed very important players in platelet thrombus formation and stability at high shear rate, whereas class II (PI3KC2α) and class III (Vps34) PI3Ks have been proven to be involved in the regulation of platelet production and functions, because they can regulate the turnover of different pools of PIP3 [61].

## 6. Conclusions

Following molecular results involving important signal transduction pathways, such as those regulated by TGF-β and SMAD, new drugs regulating erythropoiesis are now used in clinical applications. Indeed, Luspatercept has been approved in the United States and in Europe for the treatment of transfusion-dependent anemia failing treatment with ESAs in adult patients with MDS with ring sideroblasts, and for the treatment of transfusion-dependent anemia in adult patients with β-thalassemia. Luspatercept is also currently being tested in clinical trials in other subtypes of MDS (COMMAND trial), and also in other hematologic diseases associated with anemia, such as myelofibrosis [62]. As for mainly elderly and often frail MDS patients, its favorable safety profile makes it particularly suitable and manageable in this clinical setting. As for thalassemia, the use in other group of patients (NTDT and patients aged < 18 years) is still being evaluated in ongoing dedicated prospective studies.

Furthermore, some drugs, not yet in clinical trials, seem to be promising treatments. For instance, activin receptor ligand traps displayed preclinical promising efficacy in other diseases, such as Diamond–Blackfan anemia and other types of anemia [13], and Luspatercept could also be assessed in combination with other approved agents for anemia (e.g., ESAs) or with other agents targeting the TGF-β pathway (e.g., Galunisertib) to benefit from potentially synergistic or additive mechanisms of action [63,64].

As for Roxadustat, the encouraging results obtained in nephropatic, anemic patients in terms of both efficacy and safety also prompted the way to its assessment in MDS patients.

A phase III randomized, double-blind, placebo controlled (NCT03303066) study is currently ongoing. The aim of that study is to establish the efficacy and safety of Roxadustat for the treatment of anemia in patients with lower-risk MDS with low red blood cell transfusion burden. Efficacy will be measured in terms of achieving transfusion independence and increasing hemoglobin levels (≥56 consecutive days) in comparison to the baseline.

Although these trials seem to be promising in MDS therapy, drug resistance or loss of response is always possible, especially in the presence of multiple agents. That is why the identification of new therapeutic targets is essential. Within this context, a better comprehension of the role of PLC or PI3K signaling pathways, which are deeply implicated in the regulation of erythropoiesis in MDS, not only in anemia, but possibly also in thrombocytopenia, could be helpful to disclose new molecular mechanisms of erythropoiesis regulation in MDS and, possibly, become innovative promising targets for new therapies.

## Figures and Tables

**Table 1:** Clinical studies using agents that interfere with TGF-β signaling in MDS and β-thalassemia. * *p* < 0.001.

	Drug	Patients	Primary Endpoint	Outcomes	Ref
MDS	Sotatercept	Total = 74	Hematological response (HI-E)	49%	[36]
	Luspatercept	Total = 58	Hematological response (HI-E)	63%	[37]
	Luspatercept	Total = 229	Transfusion independence for ≥8 weeks during weeks 1–24		[38]
		Drug = 153 Placebo = 76		$\begin{array}{l} 38\%\\ 13\% \end{array}\}$ *	
β-thalassemia	Luspatercept	Total = 64			[39]
		NTDT = 33	Mean hemoglobin increase ≥1.5 g/dL for 14 days	58%	
		TDT = 31	Transfusion burden reduction	81%	
	Luspatercept	Total = 336	Reduction in the transfusion burden during 13–24 weeks		[40]
		Drug = 224 Placebo = 112		$\begin{array}{c} 21.4\%\\ 4.5\% \end{array}\}$ *	

## Data Availability

No new data were created or analyzed in this study. Data sharing is not applicable to this article.

## Abbreviations

ACE-011	Sotatercept
ACE-536	Luspatercept
ActRIIA	Activin receptor type IIA
ActRIIA-Fc	Fc domain of human IgG1
ActRIIB	Activin receptor type IIB
ActRIIB-Fc	Fc domain of human IgG1
ALK1-7	Activin-like receptors
Bcl-x	B-cell lymphoma-extra large
BFU-E	Erythroid burst-forming units
BMPs	Bone morphogenetic proteins
CD34+	Cluster of differentiation 34+
CFU-E	Erythroid colony-forming units
CKD	Chronic kidney disease
DAG	Dyacylglicerol
DCYTB	Duodenal Cytochrome B
DMT1	Divalent Metal Transporter 1
DOAJ	Directory of open access journals
EPC	EPO-producing cells
EPO	Erythropoietin
EPO-R	Erythropoietin receptor
ESAs	Erythropoiesis-stimulating agents
FASL	FAS ligand
GDF11	Growth/differentiating factor 11
HI-E	Hematological erythroid improvement
HIF	Hypoxia inducible factor
HIF-PHD enzymes	Hypoxia inducible factor prolyl hydrolase domain enzymes
HSC	Hematopoietic stem cells
HSP70	Heat shock protein 70
IL-3	Interleukin 3
IP3	Inositol-1,4,5-trisphospate
I-SMAD	Inhibitory small mother against decapentaplegic
JAK 2	Janus Kinase 2
LD	Linear dichroism
MAD	Mothers against decapentaplegic
MDS	Myelodysplastic syndromes
MDS-RS	Myelodysplastic syndromes with ring sideroblasts
NTDT	Non-transfusion-dependent thalassemia
PI	Phosphoinositides
PIP2	Phosphatidylinositol (4,5) bisphosphate
PIP3	PtdIns(3,4,5)P3
PI3K	Phosphatidilinositol 3-kinases
PLCs	Phospholipases C
p-SMAD	Phosphorylated small mother against decapentaplegic
ROS	Radical oxygen species
R-SMAD	Regulatory small mother against decapentaplegic
SCF	Stem cell factor
SF3B1	Splicing factor 3B subunit 1
SMAD	Small mother against decapentaplegic
STAT5	Signal Transducer and Activator of Transcription 5
TDT	Transfusion-dependent thalassemia
TEA	Treatment-emergent adverse events
TGF	Transforming growth factor
TNF	Tumor necrosis factor
WHO	World Health Organization
